# Endometrial microbiota in IVF patients: *Lactobacillus* dominance, pathogen prevalence and recurrence after treatment

**DOI:** 10.3389/fcimb.2026.1936487

**Published:** 2026-10-27

**Authors:** Diana Campos, Leire Diez-Tercero, Claudia Berenguer, Vineet Singh Raj, Claudia Díaz-López, Marta Pérez-Sánchez, Antonio Martínez-Lara, David Cotán, Mireia Sandalinas Alabert

**Affiliations:** 1 DiNA Science, Barcelona, Spain; 2 Pronacera, Sevilla, Spain

**Keywords:** endometrium, infertility, IVF, *Lactobacillus*, pathogen

## Abstract

**Introduction:**

The endometrial microbiota has been recently described as a *Lactobacillus* dominant environment and lower levels of this genus have been associated with a decrease in implantation and pregnancy rates. Moreover, the detection and overgrowth of several types of microorganisms in the female reproductive tract including bacterial, fungal and viral pathogens have been associated with complications in pregnancy such as miscarriage or preterm birth. Nevertheless, the prevalence of alterations in the endometrial microbiota of patients undergoing infertility treatment has not been thoroughly described yet.

**Methods:**

The presence of 36 microorganisms including bacteria, fungi and viruses with relevance in reproductive outcome was quantified in endometrial biopsies by Real-Time qPCR. Moreover, a second biopsy was performed in 86 patients after personalized antimicrobial and/or probiotic treatment to evaluate the efficacy of the treatment.

**Results:**

An altered microbiota was detected in 87.52% of the samples analyzed. A significantly higher presence of pathogens was detected in dysbiotic samples (*Lactobacillus* < 90%, p<0.01). More precisely, opportunistic bacterial pathogens were more frequently associated with dysbiosis (p<0.0001). *Ureaplasma parvum* and *Gardnerella vaginalis* were the most frequently detected bacterial pathogens, both individually and in combination, while *Candida spp* and Cytomegalovirus were detected in 8.2% and 1.6% of the samples. In patients that performed a second biopsy after personalized antimicrobial and probiotic treatment, a significant decrease in pathogen presence was observed (p<0.001), although *Ureaplasma parvum* showed recurrence in some patients.

**Discussion:**

Our findings suggest that imbalances in the endometrial microbiota are frequent in patients undergoing IVF treatments. Identifying these imbalances and treating them accordingly could potentially improve endometrial environment in these patients.

## 1 Introduction

In recent years, several studies have highlighted the relevance of the endometrial microbiota in reproductive outcome. Like the vaginal microbiota, several studies have described the predominance of *Lactobacillus* in the endometrium, while other bacterial species such as *Gardnerella*, *Prevotella* or *Atopobium* have also been found in lower proportions in fertile patients (Boutriq et al., 2021; Feng and Liu, 2022; Medina-Bastidas et al., 2022). Endometrial microbiota presents a lower biomass and greater microbial diversity compared to the vaginal microbiota (Gao et al., 2024). High *Lactobacillus* levels contribute to reproductive health by producing lactic acid and hydrogen peroxide, modulating the local environment and preventing pathogen colonization. Additionally, *Lactobacillus* metabolites stimulate antimicrobial peptides and anti-inflammatory cytokines, creating a favorable environment for implantation and pregnancy (Łaniewski et al., 2020).

However, this concept of a stable “*Lactobacillus*-dominated endometrium” has been increasingly questioned. Some studies suggested that the frequently reported predominance of *Lactobacillus* in endometrial samples may partly reflect contamination from vaginal *Lactobacillus* species during sample collection rather than a true endometrial microbial signature (Winters et al., 2019). Similarly, other studies focusing on metabolically active microorganisms, reported that *Lactobacillus* does not appear to dominate the endometrium of healthy women (Sola-Leyva et al., 2021). Due to the variability of published results in different studies, it has not yet been possible to determine exactly what constitutes a normal endometrial microbiome.

Despite this heterogeneity, in IVF populations *Lactobacillus* dominance has still been associated with improved reproductive outcomes. Reduced *Lactobacillus* abundance below defined thresholds (typically <90–80%) and microbial dysbiosis have been correlated with lower implantation, pregnancy, and live birth rates, as well as increased miscarriage risk (Moreno et al., 2016; Koedooder et al., 2019a, b; Kyono et al., 2019). Opportunistic bacterial overgrowth associated with vaginosis could also interfere with the implantation process and with the achievement of a clinical pregnancy (Haahr et al., 2016; Diaz-Martínez et al., 2021; Ichiyama et al., 2021). In addition, the presence of certain microorganisms with potential pathogenicity in the endometrium such as *Ureaplasma* or *Mycoplasma* have been associated with infertility, as well as implantation failures, miscarriage and a higher risk of preterm birth (Moreno et al., 2018; Ravel et al., 2021; Toson et al., 2022).

Recent studies also highlight the impact of fungi and viruses on the reproductive tract. Candida infections affect approximately 75% of women at least once in their lifetime, with 20% being asymptomatic. Several case reports have suggested that intrauterine infection with *Candida* species (*albicans* and *glabrata*) might be associated with higher rates of preterm birth, as well as miscarriage (Ito et al., 2013; Alfei et al., 2014; Kusanovic et al., 2018; Yusoff et al., 2022). However, further research is needed to confirm its effects on implantation and pregnancy outcomes.

Viruses like Cytomegalovirus (CMV) and Herpes Simplex Virus (HSV1 and HSV2) are also associated with adverse pregnancy outcomes and negative effects on the fetus. CMV infections during the preconception period show a 5.5% vertical transmission rate without newborn effects, while periconception infections increase the transmission rate to 21% and foetal involvement to 28.8%, leading to conditions like hearing loss and microcephaly (Chatzakis et al., 2020; Kirschen and Burd, 2023). Regarding HSV1-2, infection with this type of virus has been associated with increased risk of miscarriage, preterm labor, and congenital and neonatal herpes (Farsimadan and Motamedifar, 2021). Infections during the first two trimesters are linked to spontaneous miscarriage and fetal malformations, while third-trimester infections may result in preterm birth, antenatal hypotrophy, hypoxia, and fetal death. A recent study also described that recurrent HSV1 also caused complications during childbirth and respiratory distress syndrome and cerebral ischemia in newborns (Andrievskaya et al., 2022).

The aim of this report is to determine the prevalence of different endometrial microbiota profiles in infertile patients, focusing on *Lactobacillus* dominance and the presence of pathogens (bacteria, fungi, viruses). Moreover, the effect of antimicrobial treatment in the composition of endometrial microbiota of patients with a pathogenic profile that performed a second biopsy after treatment was analyzed.

## 2 Materials and methods

### 2.1 Population

A multicenter study, involving 1619 patients treated at 58 IVF clinics and undergoing endometrial analysis with the ES-META microbiota genomic test in 2022, included participants aged over 18 with a history of previous implantation failure, unexplained miscarriages, infertility of unknown origin, recurrent vaginosis and/or endometritis/endometriosis, and a previous clinical history of autoimmune or inflammatory disease. Data regarding the age, BMI, indication and type of cycle performed by patients prior to the endometrial biopsy can be found in **Table 1**.

**Table 1:** Descriptive characteristics of the patients included in the study.

Patient characteristics	Total	Implantation failure (IF)	Recurrent implantation failure (RIF)	Miscarriage	Other indications	p
n	1619	853	289	201	276	
Age	40.17 ± 4.36	40.33 ± 4.40	40 ± 4.34	40.01 ± 4.56	39.99 ± 4.15	0.67
Body Mass Index (BMI)	20.74 ± 4.12	20.91 ± 4.27	20.48 ± 3.93	20.76 ± 3.77	20.55 ± 4.06	0.64
Day of cycle	19.25 ± 4.44	19.31 ± 4.5	19.22 ± 4.14	19.56 ± 4.79	18.89 ± 4.24	0.56
Type of hormonal cycle						
*Natural LH*	396 (24.46%)	165 (19.34%)	58 (20.07%)	91 (45.27%)^a^	82 (29.71%)	
*Natural hCG*	85 (5.25%)	43 (5.04%)	23 (7.96%)	6 (2.99%)^b^	13 (4.71%)	
*Hormone Replacement Therapy (HRT)*	1138 (70.29%)	645 (75.62%)	208 (71.97%)	104 (51.74%) ^a^	181 (65.58%)	

^a^ p<0, 01 vs total, IF, RIF and Other indication columns.

^b^ p=0, 036 vs RIF column.

A confirmatory biopsy after performing antimicrobial and probiotic treatment was performed on 86 patients due to the detection of strict pathogenic species (n=39) and due to the overgrowth of opportunistic bacteria or a reduction in the *Lactobacillus* % (N = 47). Treatments were administered orally or vaginally and selected by clinicians following standard antimicrobial guidelines.

### 2.2 Sample collection

The specimen utilized in this study was an endometrial biopsy, collected by a gynecologist using a standard procedure. Endometrial biopsies were recommended to be conducted within a mock cycle—either after 7 days of the LH surge or stimulation with hCG in natural cycles, or after 5 days of progesterone supplementation in hormone-replacement therapy cycles.

The endometrial biopsies, approximately 30 mg each, were extracted from the uterine fundus using a Pipelle catheter (Gynetics, Namont-Achel, Belgium). Special attention was given to avoid the collection of mucous tissue or coagulated blood. Prior to catheter insertion, visible cervical mucus and excess blood, when present, were gently removed by brief localized dabbing with sterile gauze, avoiding contact with the vaginal walls to minimize the risk of vaginal contamination. Subsequently, the collected tissue was placed in a 2mL CryoTube^®^ (Nunc, Roskilde, Denmark) containing 1.2 mL of RNA Keeper Tissue stabilizer (Vazyme, China). The samples were then stored at 4 °C for a minimum of 4 hours before undergoing further processing.

### 2.3 Endometrial microbiota analysis

A total of 1713 tests were performed including 94 re-biopsies for reanalysis purposes.

Total DNA from biopsies was extracted using the ReliaPrep Blood gDNA Miniprep kit System (Promega, USA), following the manufacturer’s instructions, including specific protocol modifications provided for enhanced microbial DNA isolation. The quality of the total DNA was assessed using a Multiskan SkyHigh Photometer (Thermo Fisher Scientific, USA) by analyzing concentration (ng/μl) and quality ratios (260/280 and 260/230 nm).

Preamplification was performed to increase sensitivity and compensate for variable amplification efficiencies among targets, ensuring low-abundance microbial DNA and less efficient targets are adequately represented. For preamplification, DNA was combined with a master mix (Standard BioTools, USA) prepared with Preamp master mix, Pooled TaqMan assay mix, and PCR-certified water. Samples were then pre-amplified using a T100 thermocycler (Bio-Rad, USA) following the manufacturer’s instructions. Next, the product was purified using a mix of Exonuclease I Reaction Buffer (New England Biolabs, USA) and Exonuclease I (20 U/μl) (New England Biolabs, USA), which were added to each preamplified sample. After that, a T100 thermocycler program was carried out following manufacturer’s specifications.

Finally, microbiome testing employed microfluidic RT-PCR using the Biomark X platform (Standard BioTools, USA) with TaqMan Fast Advanced Master Mix (Thermo Fisher Scientific, USA) gene expression commercial probes for 36 microorganisms (**Supplementary Table S1**), according to the manufacturer’s guidelines. This included microorganisms with pathogenic potential, whether they are opportunistic or strict pathogens. Microorganisms were considered pathogenic according to literature-based classification, when they interfere negatively with the outcome of the IVF procedures, as certain microorganisms may alter the endometrial microenvironment by inducing an inflammatory response, which may impair embryo implantation or increase the risk of adverse pregnancy outcomes.Negative controls (ultrapure distilled water free of DNase/RNase (Invitrogen, USA) and DNA extracted from the preservative solution) were included at the DNA extraction, pre-amplification, and final microfluidic PCR steps to monitor potential contamination throughout the workflow.

### 2.4 RT-PCR data analysis

The data underwent initial examination using graphic representations that illustrated the amplification profile for each probe. Additionally, the Quality Threshold of the PCR system analysis software was assessed for each amplification. In the microbiota analysis, DNA standard curves were established using amplification data representing various genomic index values for each microorganism. Ct values obtained from each assay during data analysis were then transformed into genomic indexes through extrapolation on the respective curves. Subsequently, each genomic index underwent analysis in relation to its reference range value and the relative abundance was calculated extrapolating the colony forming units from the genomic index.

### 2.5 Statistical analysis

The number of samples with increasing *Lactobacillus* percentages and with detection of pathogenic species were quantified and percentages were calculated concerning the total number of samples or relative to the total pathogenic samples. The statistical analysis was performed using GraphPad InStat (v3.06, 2003; GraphPad Software, Inc) and R (v4.3.1). To determine whether the differences between patients were significant or not, depending on data obtained, the Chi-square test of independence or Fisher’s exact test were used. The comparison of the *Lactobacillus* percentage in patients with two biopsies was performed using Wilcoxon matched paired test. Paired analysis comparing the reduction in pathogen detection between 1^st^ and 2^nd^ biopsies was performed using McNemar’s test. Statistical significance was accepted at p<0.05.

## 3 Results

### 3.1 Eubiotic/dysbiotic profile

Test results were categorized based on *Lactobacillus* relative abundance and the presence of microorganisms with potential pathogenicity (opportunistic or strict). Samples with over 90% *Lactobacillus* were considered eubiotic (*Lactobacillus*-dominant), while those with lower percentages were considered dysbiotic (non-*Lactobacillus*-dominant) (Moreno et al., 2016).

Analysis of the first endometrial microbiota test performed in all the patients revealed that most samples were dysbiotic (**Table 2**), with 37.61% (609 samples) showing *Lactobacillus* relative abundance below 10% (**Figure 1**). The average of the *Lactobacillus* relative abundance in eubiotic and dysbiotic groups was 96.77% ± 2.95% and 26.66% ± 28.83%, respectively.

**Table 2:** Distribution of the different profiles regarding Lactobacillus dominance (eubiosis/dysbiosis) and presence of pathogens.

Profile	Sample number	Percentage
TOTAL	1619	100.00%
EUBIOSIS	271	16.74%
Non-pathogenic eubiosis	202	12.48%
Pathogenic eubiosis	69	4.26%
DYSBIOSIS	1348	83.26%
Non-pathogenic dysbiosis	880	54.35%
Pathogenic dysbiosis	468	28.91%

**Figure 1:**
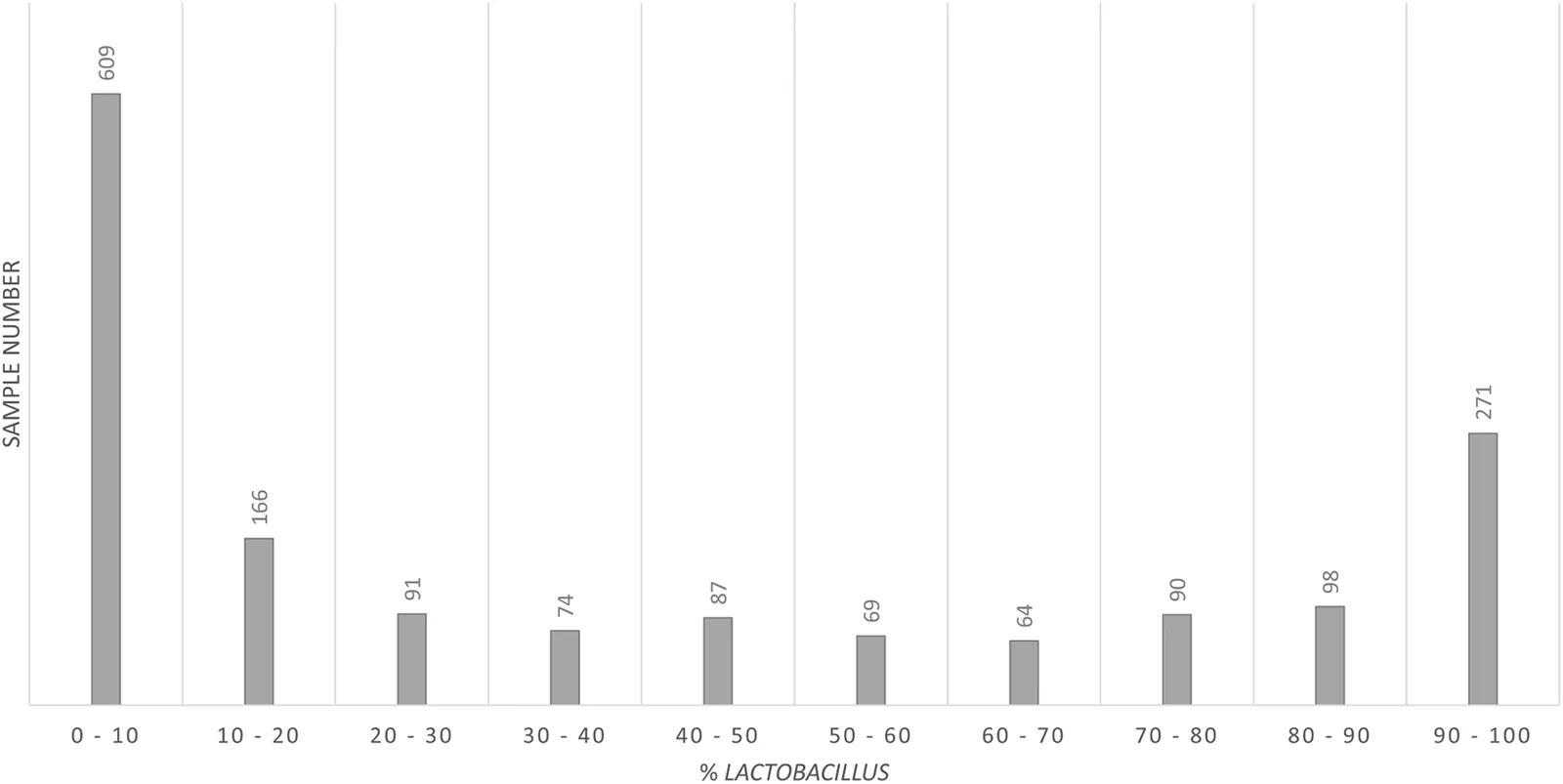
Distribution of samples regarding the Lactobacillus percentage in the 1^st^ biopsy.

No statistically significant associations were observed between microbial composition and the variables evaluated, including BMI and the day of the menstrual cycle at sample collection. The only exception was a statistically significant difference in the day of sample collection between non-pathogenic dysbiotic and eubiotic patients; however, this finding was not considered clinically relevant. No statistically significant differences were observed in the rates of eubiosis/dysbiosis or pathogenicity comparing the data between IVF clinics.

### 3.2 Pathogen presence

Samples were classified as either pathogenic or non-pathogenic, depending on the detection or overgrowth of various bacteria, viruses, fungi and protozoan, considering as pathogens both opportunistic and strict microorganisms with pathogenic potential. At least one pathogen was detected in 33.17% of the samples, with most pathogenic samples being dysbiotic (87.15%). Dysbiotic samples showed a significantly higher proportion of pathogens than eubiotic samples (p=0.003943). (**Table 2**).

Among the pathogenic bacteria, *Ureaplasma parvum* and *Gardnerella vaginalis* were identified most frequently, appearing in both eubiotic and dysbiotic samples. In eubiotic samples, *Ureaplasma parvum* was the predominant pathogen (**Table 3**), although no statistically significant differences were found compared to dysbiotic samples (p = 0.2112). Conversely, *Gardnerella vaginalis* was more common in dysbiotic than in eubiotic samples (p <0.0001).

**Table 3:** Distribution of the different types of pathogens (bacterial, fungal, protozoan and viral) detected in endometrial samples.

Microorganism	General		Eubiosis (% *Lactobacillus* ≥90%)		Dysbiosis (% *Lactobacillus* < 90%)		
Type of pathogen	n	% vs sample total	n	% vs eu total	n	% vs dis total	p value
Bacterial pathogens							
*Ureaplasma parvum*	149	9.2%	31	.11.44%	118	8.75.%	0.2112
*Mycoplasma hominis*	14	0.9%	3	1.11.%	11	0.82.%	0.7163
*Ureaplasma urealyticum*	17	1.1%	3	1.11.%	14	1.04.%	1.000
*Chlamydia trachomatis*	3	0.2%	0	0.00%	3	0.22.%	1.000
*Neisseria gonorrhoeae*	1	0.1%	0	0.00%	1	0.07.%	1.000
*Gardnerella vaginalis*	165	10.2%	8	2.95.%	157	11.65.%	<0.0001
*Streptococcus* spp	31	1.9%	1	0.37.%	30	2.23.%	0.0478
*Mobiluncus* spp	15	0.9%	1	0.37%	14	1.04%	0.4891
*Actinomyces*	27	1.7%	3	1.11.%	24	1.78.%	0.6045
*Fusobacterium*	3	0.2%	0	0.00%	3	0.22.%	1.000
Fungal pathogens							
*Candida* spp	132	8.2%	12	4.43.%	120	8.90.%	0.0194
*Candida albicans*	17	1.1%	5	1.85.%	12	0.89.%	0.1866
*Candida glabrata*	3	0.2%	0	0%	3	0.22.%	1.000
Viral pathogens							
Cytomegalovirus	26	1.6%	7	2.58.%	19	1.41.%	0.1844
Herpes simple virus 1	1	0.1%	0	0.00%	1	0.07.%	1.000
Protozoo							
*Trichomonas vaginalis*	2	0.1%	0	0.00%	2	0.15.%	1.000

The relative abundance was calculated concerning the total number of samples or relative to the total of eubiotis or dysbiotic samples within each group for the “Eubiosis” and “Dysbiosis” columns. Statistical analysis was performed comparing the number of pathogens of each type in eubiotic and dysbiotic groups.

Viral and fungal pathogens were also identified in endometrial samples, with *Candida* spp. detected in 8.2% of samples and viruses (specifically, Cytomegalovirus and Herpes Simplex Virus 1) in 1.7%. *Candida* spp. was significantly more prevalent in dysbiotic samples (p=0.0194), while viruses were more common in eubiotic ones, though this difference was not statistically significant.

Some samples contained multiple pathogens (**Supplementary Table S2**). In the eubiotic group (**Supplementary Table S3**), the most frequent combinations were *Ureaplasma parvum* with *Candida* spp. (4 samples) and *Ureaplasma* spp. with Cytomegalovirus (2 samples). In dysbiotic samples (**Supplementary Table S4**), the most frequent combinations included *Ureaplasma parvum* with *Gardnerella vaginalis* (11 samples), *Ureaplasma parvum* with *Candida* spp. (11 samples), and *Gardnerella vaginalis* with *Candida* spp. (7 samples). Additionally, eleven samples contained three pathogens, with five of them featuring a combination of *Ureaplasma* and *Gardnerella* with *Candida* spp.

The pathogen types (strict, opportunistic, fungi, virus and protozoa) differed between eubiotic and dysbiotic samples (**Table 4**). Eubiotic samples had a higher prevalence of strict pathogens, while dysbiotic samples showed a significantly higher presence of opportunistic bacteria (p<0.0001). Viral pathogens were more common in eubiotic samples, though the difference was not significant (p=0.1993). Fungi were evenly distributed between groups (p=0.0843).

**Table 4:** Proportion of the different types of pathogens (bacterial [strict and opportunistic], fungal, protozoan and viral) detected in endometrial samples.

Type of pathogens	Total		Eubiosis		Dysbiosis		
	n	% vs total	n	% vs eu total	n	% vs dys total	p value
Strict bacterial pathogen	186	11.4%	37	13.65%	149	11.05%	0.3030
Opportunistic bacterial pathogen	241	14.9%	13	4.80%	228	16.91%	<0.0001
Fungi	152	9.6%	17	6.27%	135	10.01%	0.0843
Viruses	27	1.8%	7	2.58%	20	1.48%	0.1993
Protozoa	2	0.1%	0	0%	2	0.15%	1.000

The relative abundance was calculated concerning the total amount of samples or relative to the total of eubiotic or dysbiotic samples within each group for the “Eubiosis” and “Dysbiosis” columns. Statistical analysis was performed comparing the number of pathogens of each type in eubiotic and dysbiotic groups.

When analyzing the presence of pathogens according to the type of cycle the patients were on (Natural LH or HRT) (**Supplementary Table S5**), no significant differences were observed.

### 3.3 Second biopsies

A subset of patients (n = 86) underwent second or third biopsies post-treatment for altered microbiota results (**Supplementary Figure S1**). A new biopsy after antimicrobial and probiotic treatment was recommended in cases classified as pathogenic. However, a new biopsy was performed in some patients in the dysbiotic group and with a non-pathogenic result after probiotic treatment. The average time between biopsies was 79.35 ± 36.25 days, although this time period was not necessarily correlated with the duration of probiotic treatment, which was variable among patients. Among those with two biopsies, the percentage of samples with pathogens decreased significantly in both eubiotic and dysbiotic groups (**Table 5**, p < 0.001). Moreover, analyzing the distribution of the samples regarding the percentage of *Lactobacillus*, a decrease was observed in the number of samples with low *Lactobacillus* percentage (<10%), while there was an increase in the number of samples with higher *Lactobacillus* percentages (**Supplementary Figure S2**), although paired statistical analysis showed no significant differences (p=0.0909).

**Table 5:** Distribution of the different profiles regarding *Lactobacillus* dominance (eubiosis/dysbiosis) and presence of pathogens of the same patients in the 1^st^ biopsy and in samples taken after treatment.

Biopsy timing	%EU	% EU PAT	% EU NO PAT	%DIS	% DIS PAT	% DIS NO PAT
Pre-treatment (1st biopsy)	15.58%	12.99%	2.60%	84.42%	71.43%	12.99%
Post-treatment (2^nd^ biopsy)	20.78%	.1.30%	19.48%	79.22%	15.58%	63.64%

Of these 86 patients (77 with 2 biopsies and 9 with 3 biopsies, **Supplementary Figure S1**), 15 (17.4%) underwent a second biopsy despite a non-pathogenic first result. Pathogens were detected in the 2^nd^ biopsy of two of these patients (Cytomegalovirus and *Gardnerella vaginalis* overgrowth). *Lactobacillus* levels decreased or remained unchanged in 9 cases and increased in 6. From the 77 patients with two biopsies, a significant decrease was observed in pathogen detection (p<0.0001).

The remaining 71 patients (82.6%) had a second biopsy after a pathogenic first result (**Supplementary Table S6**). Following treatment, 54 (76.06%) had non-pathogenic second results, while 17 (23.94%) still showed pathogens, either the same or different ones. Among the 17 persistent cases, 8 retained the same pathogen (most commonly *Ureaplasma parvum*, in 6 cases). The other 9 patients had different pathogens in the 2 biopsies, with 4 patients having a strict pathogen in the 1^st^ biopsy and an opportunistic pathogen in the 2^nd^ one and 4 patients had different opportunistic bacteria in both samples.

9 patients underwent a third biopsy. Of these, 3 had non-pathogenic results throughout, while 6 had persistent pathogens in the first 2 samples. In 4 cases, no pathogens were detected in the third sample. Interestingly, one patient with persistent *Ureaplasma parvum* was treated twice with doxycycline and, only after her sexual partner was treated after the second biopsy, this pathogen was no longer detected. However, 2 patients had pathogenic third biopsies, involving a viral pathogen (Cytomegalovirus) and an opportunistic bacterium (*Actinomyces*), respectively.

### 3.4 Microbiota across patient groups

Comparison of the microbiota between patients with implantation failure (IF: 1–2 failed embryo transfers), recurrent implantation failure (RIF: ≥3 failed transfers), and patients with previous miscarriages revealed no significant differences in eubiosis/dysbiosis rates or the proportion of pathogenic samples in each group (**Supplementary Table S7**). Similarly, the types and proportions of pathogens detected did not differ among these groups (data not shown). When assigning patients to the respective groups, the primary indication for performing the test was considered. Patients with more than one indication or if the primary indication could not be determined were classified into the “Other indications” group.

## 4 Discussion

The vaginal microbial composition has been extensively studied, with a healthy state typically associated with a *Lactobacillus*-dominant (LD) environment. A reduction in *Lactobacillus* relative abundance, combined with the overgrowth of opportunistic bacteria or fungi and the presence of certain pathogens, can lead to conditions such as bacterial vaginosis, candidiasis, or vaginitis. Recent research underscores the importance of an LD endometrial environment, as it correlates with higher implantation and ongoing pregnancy rates (Moreno et al., 2022; Gao et al., 2024; Su et al., 2024). Moreover, a non-LD endometrium has been associated with increased levels of pro-inflammatory markers compared with LD patients, suggesting adverse reproductive outcomes (Cela et al., 2022; Odendaal et al., 2024).

The threshold of ≥90% *Lactobacillus* relative abundance to define endometrial eubiosis was used because it represents the most widely used and historically established criterion in studies evaluating the clinical relevance of the endometrial microbiome. This cutoff was originally proposed by Moreno et al. (2016), who classified endometrial microbiota as *Lactobacillus*-dominated (LDM; >90% *Lactobacillus* spp.) or non-*Lactobacillus*-dominated (NLD; <90%), and reported significantly poorer implantation, clinical pregnancy, ongoing pregnancy, and live birth outcomes in patients with NLD microbiota (Moreno et al., 2016). The results of a study performed by Cela et al., 2022, showed significantly higher levels of some pro-inflammatory interleukins in patients with endometrial dysbiosis (Cela et al., 2022). Other several independent studies and reviews have adopted the same threshold, supporting its use as the reference definition for endometrial eubiosis (Hashimoto and Kyono, 2019; Stoyancheva et al., 2025).

Although alternative definitions have been proposed, including a threshold of ≥80% *Lactobacillus*, and some studies have questioned the biological validity of a strict threshold, the ≥90% remains the most widely validated and systematically applied threshold in the scientific literature (Kyono et al., 2018). However, further studies with clinical outcomes are needed to determine whether the abundance of *Lactobacillus* might also be better interpreted as a continuous variable rather than establishing a specific cutoff.

The results obtained in this study show that most IVF patients had a dysbiotic endometrial environment, with a relative abundance of *Lactobacillus* lower than 10% in almost half of them. Previous studies have reported conflicting results in endometrial fluid and tissue of IVF patients, describing different rates of Eubiosis and Dysbiosis, and the evidence regarding the prevalence of a *Lactobacillus* dominated endometrial environment in IVF patients is limited (Kyono et al., 2019; Reschini et al., 2022; Kadogami et al., 2023).

Pathogenic species were predominantly observed in dysbiotic samples, with *Ureaplasma* spp. and *Gardnerella vaginalis* being the most frequently detected, followed by *Candida* spp (**Table 3**). Several studies have reported rates of detection of *Ureaplasma* species in the endometrium ranging between 10 and 22.2%, which are slightly higher compared to this study (Cicinelli et al., 2012; Takimoto et al., 2023). Moreover, rates between 1 and 40.7% have been previously reported in endometrial samples in the case of *Gardnerella vaginalis* (Cicinelli et al., 2012; Takimoto et al., 2023). Its overgorwth in the female reproductive tract is also associated with complications like vaginosis, preterm rupture of membranes and adverse pregnancy outcomes (Silva et al., 2014; Kacerovsky et al., 2021; Savicheva et al., 2023; Munch et al., 2024).

Regarding the presence of *Candida* in the female reproductive tract, approximately 10–15% of asymptomatic women carry this microorganism in their reproductive tract, and 70–75% experience vulvovaginal candidiasis at least once in their lifetime, being the main cause of vaginitis after bacterial vaginosis (Gonçalves et al., 2016). However, there are few studies reporting its presence in the endometrium. Jain et al. determined that the presence of *Candida* spp was lower in the endometrium compared with the cervix and the vagina (2%, 7% and 11%, respectively) (Jain et al., 2023) Other studies have reported similar rates in vaginal samples, and have suggested a possible correlation between the presence of *Candida* and intrauterine adhesions (Savicheva et al., 2023; Zhao et al., 2023). Recurrent pregnancy loss (RPL) patients also experience higher rates of candidiasis and bacterial vaginosis, suggesting a possible interference in fertility caused by fungi (Peuranpää et al., 2022). Nevertheless, the precise effect of *Candida* infection in the reproductive outcome in IVF patients remains to be understood and more studies are required.

Remarkably, viral presence was detected in few samples (more precisely, Cytomegalovirus, **Table 3**), consistent with earlier studies reporting 1.5–4% prevalence in vaginal and endometrial samples (Jain et al., 2023; Savicheva et al., 2023). This suggests a low prevalence of viral load in the female reproductive tract. However, due to the possible vertical transmission of Cytomegalovirus and the severe effects of congenital infection, performing this type of analysis could provide useful information for the clinical management of IVF patients (Manicklal et al., 2013). In short, few studies have determined the prevalence of these species in endometrial samples of patients with recurrent miscarriage or recurrent implantation failure and therefore, more evidence is needed to determine the proportion of each pathogenic species in this type of population.

Multiple opportunistic or fungal pathogens coexisted in some patient samples, with combinations such as *Ureaplasma parvum* + *Gardnerella vaginalis*, *Ureaplasma parvum* + *Candida* spp., and *Gardnerella vaginalis* + *Candida* spp. being most prevalent. These findings align with reports of pathogen co-occurrence in vaginal samples (Maraki et al., 2018; Rak et al., 2022). Some studies have found a correlation between the detection of microorganisms associated with bacterial vaginosis and STIs and the overgrowth of other pathogens (Rak et al., 2022), though the exact mechanisms remain unclear (Maraki et al., 2018; Rak et al., 2022).

A notable association was observed between the type of pathogen (strict or opportunistic) and the percentage of *Lactobacillus*. Strict pathogens, like *Ureaplasma parvum*, were the most prevalent pathogens in LD environments, while opportunistic pathogens, such as *Gardnerella vaginalis*, were linked to dysbiotic conditions (**Table 3**). *Ureaplasma* spp is considered a sexually transmitted microorganism that has been associated with infertility, preterm labor and neonatal morbidity and its ability to colonize the female reproductive tract seems to be independent of *Lactobacillus* dominance (Hong et al., 2021; Liu et al., 2022; Rittenschober-Boehm et al., 2024). Its detrimental effects may stem from inflammation and cellular damage caused by enzymatic and protein activity rather than microbiota alterations (Ireland and Keelan, 2014; Lal et al., 2017; Garza et al., 2021; Liu et al., 2024).

On another note, *Lactobacillus* species have been described to generate a protective environment against the overgrowth of opportunistic pathogens through the release of several molecules (Tachedjian et al., 2017; Colautti et al., 2022). In fact, it has been previously described that there is a negative correlation between *Lactobacillus* spp and bacterial species associated with bacterial vaginosis (Lozano et al., 2021).

However, external factors like antibiotics or environmental factors can deplete *Lactobacillus* populations, enabling opportunistic bacteria to proliferate (Kwon and Lee, 2022). In this sense, the use of probiotics that contain *Lactobacillus* species has been associated with a decrease in the Nugent score and an increase in the presence of *Lactobacillus* in the vagina, suggesting that this type of treatment could be effective in the restoration of the microbiota in dysbiotic patients (Fernández et al., 2021; Ansari et al., 2023; Pendharkar et al., 2023; Wu et al., 2024).

Patients with pathogen-positive first biopsies underwent subsequent biopsies following antimicrobial and probiotic treatment. In most cases, pathogens were not detected after treatment, and *Lactobacillus* relative abundance increased, resulting in higher rates of eubiosis and a lower proportion of samples with very low relative abundance of *Lactobacillus* (<10%) (**Supplementary Figures S1**, **S2** and **Table 5**). However, variability in treatment duration and probiotic formulations may have influenced these outcomes. Some studies have suggested that probiotic treatments of at least 3–6 months have a significant effect in increasing the relative abundance of *Lactobacillus* and have been associated with an improvement in pregnancy outcome (Fernández et al., 2021; López-Moreno and Aguilera, 2021; Mei and Li, 2022; Iwami et al., 2023).

On the other hand, the disruption of the reproductive microbiota, especially associated with antibiotics, is a major risk factor for bacterial pathogen colonization and for those growing bacterial resistance towards antibiotics. Broad range antibiotics, which are frequently used in clinical settings, do not discriminate among different types of bacteria and a favorable physiological environment is created for pathogens, consequently promoting their growth. In this sense, the overgrowth/presence of pathogenic bacteria was detected in the second biopsy of 17 patients and 8 still had the same type of pathogen, suggesting a possible antibiotic resistance or a reinfection due to a possible lack of intervention in the sexual partner of the patient. The pathogen that persisted most frequently was *Ureaplasma parvum* (6 patients). This microorganism is known to develop resistance to antibiotics, including macrolides, tetracyclines, and fluoroquinolones, with resistance rates varying regionally (Liu et al., 2024). Furthermore, the overgrowth of opportunistic pathogens was detected in 8 patients, suggesting that even though the recommended treatment reduced the bacterial load of the target microorganism, the ecological niche was then colonized by other species instead of *Lactobacillus*.

## 5 Conclusion

The findings in this article highlight that a high proportion of patients treated in IVF clinics present an imbalanced endometrial microbiota, with either overgrowth of certain pathogens and/or a low relative abundance of *Lactobacillus* species. Therefore, evaluating the endometrial microbiota, including pathogen presence, could potentially provide relevant information for patients with specific reproductive challenges, such as recurrent implantation failure or miscarriage, since these imbalances have been associated with complications in embryo implantation and pregnancy in literature. Moreover, differences in the composition of the endometrial microbiota were found in patients with a second biopsy performed after antimicrobial or probiotic treatment, with significantly lower presence of pathogenic species after treatment. Nevertheless, further studies using an untreated group and reproductive outcome data are required to evaluate the efficacy of treatments in dysbiotic and/or pathogenic profiles, as well as evaluating the composition of the microbiota in the sexual partners to avoid possible reinfections in microorganisms that can be sexually transmitted.

## Data Availability

The original contributions presented in the study are included in the article/**Supplementary Material**. Further inquiries can be directed to the corresponding author.
