# Supplementary material for: Endometrial microbiota in IVF patients: Lactobacillus dominance, pathogen prevalence and recurrence after treatment
Source: Front Cell Infect Microbiol. 2026 Oct 27;16:1936487. doi: 10.3389/fcimb.2026.1936487 (PMC13748442; doi:10.3389/fcimb.2026.1936487)
Supplement: Supplementary file 1 [file SupplementaryFile1.docx]

**Supplementary material**

Table S1. *Types of microorganisms analyzed in endometrial samples.*

| **Acid lactic bacteria** | **Non-acid lactic bacteria** | **Opportunistic pathogens** | **Strict Bacterial pathogens** | **Fungi** | **Virus** |
| --- | --- | --- | --- | --- | --- |
| *Lactobacillus* spp. | *Bacteroides* spp. | *Gardnerella vaginalis* | *Chlamydia trachomatis* | *Candida* spp. | Herpes simplex virus 1 |
| *Lactobacillus crispatus* | *Prevotella* spp. | *Streptococcus* spp. | *Neisseria gonorrhoeae* | *Candida albicans* | Herpes simplex virus 2 |
| *Lactobacillus gasseri* | *Staphylococcus* spp. | *Mobiluncus* spp. | *Mycoplasma hominis* | *Candida glabrata* | Cytomegalovirus |
| *Lactobacillus jensenii* | *Enterobacteriaceae* | *Actinomyces* spp. | *Mycoplasma genitalium* |  | Varicella-zoster Virus |
| *Lactobacillus iners* | *Sneathia amnii* | *Fusobacterium* spp. | *Ureaplasma parvum* |  |  |
| *Bifidobacterium* spp. | *Corynebacterium* spp. |  | *Ureaplasma urealyticum* |  |  |
| *Enterococcus* spp. |  |  | *Trichomonas vaginalis* |  |  |
| *Atopobium vaginae* |  |  | *Haemophilus drucreyi* |  |  |
|  |  |  | *Chlamydia trachomatis* (LGV) |  |  |
|  |  |  | *Treponema pallidum* |  |  |

Table S2. *Distribution of the different types of pathogens (bacterial, fungal, protozoan and viral) detected in endometrial samples, either alone or in combination with other pathogens. The percentages were calculated relative to the total amount of samples with each type of pathogen in the eubiotic or dysbiotic group.*

|  | Eubiosis | | | | | Dysbiosis | | | | |
| --- | --- | --- | --- | --- | --- | --- | --- | --- | --- | --- |
|  | Only 1 pathogen | | More than 1 pathogen | | Total | Only 1 pathogen | | More than 1 pathogen | | Total |
| Pathogen | n | % vs eu (vs total) | n | % vs eu (vs total) | n | N | % vs dys (vs total) | n | % vs dys (vs total) | n |
| *Ureaplasma parvum* | 24 | 8.86.% (77%) | 7 | 2.58.% (23%) | 31 | 77 | .5.71% (65%) | 41 | 3.04.% (35%) | 118 |
| *Mycoplasma hominis* | 1 | .0.37% (33%) | 2 | 0.74.% (67%) | 3 | 3 | 0.22% (27%) | 8 | 0.59.% (73%) | 11 |
| *Ureaplasma urealyticum* | 1 | 0.37.% (33%) | 2 | 0.74.% (67%) | 3 | 7 | 0.52.% (50%) | 7 | 0.52.% (50%) | 14 |
| *Chlamydia trachomatis* | 0 | - | 0 | - | 0 | 1 | 0.07% (33%) | 2 | 0.15.% (67%) | 3 |
| *Trichomonas vaginalis* | 0 | - | 0 | - | 0 | 1 | 0.07% (50%) | 1 | 0.07.% (50%) | 2 |
| *Neisseria gonorrhoeae* | 0 | - | 0 | - | 0 | 1 | 0.07% (100%) | 0 | 0% (0%) | 1 |
| *Gardnerella vaginalis* | 7 | 2.58.% (88%) | 1 | 0.37.% (13%) | 8 | 124 | 9.20.% (79%) | 33 | 2.45.% (21%) | 157 |
| *Streptococcus* spp. | 1 | .0.37% (100%) | 0 | 0% (0%) | 1 | 22 | 1.63.% (73%) | 8 | 0.59.% (27%) | 30 |
| *Mobiluncus* spp. | 1 | 0.37.% (100%) | 0 | 0% (0%) | 1 | 9 | 0.67.% (64%) | 5 | 0.37.% (36%) | 14 |
| *Actinomyces* | 3 | 1.11.% (100%) | 0 | 0% (0%) | 3 | 19 | 1.41.% (79%) | 5 | 0.37.% (21%) | 24 |
| *Fusobacterium* spp. | 0 | - | 0 | - | 0 | 1 | 0.07% (33%) | 2 | 0.15.% (67%) | 3 |
| *Candida* spp. | 8 | 2.95.% (67%) | 4 | 1.48.% (33%) | 12 | 86 | 6.38.% (72%) | 34 | 2.52.% (28%) | 120 |
| *Candida albicans* | 5 | .1.85% (100%) | 0 | 0% (0%) | 5 | 8 | 0.59.% (67%) | 4 | 0.30.% (33%) | 12 |
| *Candida glabrata* | 0 | - | 0 | - | 0 | 1 | 0.07% (33%) | 2 | 0.15.% (67%) | 3 |
| Cytomegalovirus | 5 | 1.85.% (71%) | 2 | 0.74% (29%) | 7 | 13 | 2.8% (68%) | 6 | 0.45.% (32%) | 19 |
| Herpes simplex virus 1 | 0 | - | 0 | - | 0 | 0 | 0% (0%) | 1 | 0.07.% (100%) | 1 |

*Table S3. Combinations and accounting of pathogens found in eubiotic samples.*

| Eubiosis | *U. parvum* | *M. hominis* | *U. urealyticum* | *Gardnerella* | *Candida spp* |
| --- | --- | --- | --- | --- | --- |
| *U. parvum* | - | 1 | - | 1 | 4 |
| *M. hominis* | - | - | 1 | - | - |
| Cytomegalovirus | 1 | - | 1 | - | - |

*Table S4. Combinations and accounting of pathogens found in dysbiotic samples with 2 different pathogens. Most frequently detected combinations are highlighted in green.*

| Dysbiosis | *Ureaplasma parvum* | *Mycoplasma hominis* | *Ureaplasma urealyticum* | *Chlamydia trachomatis* | *Trichomonas vaginalis* | *Neisseria gonorrhoeae* | *Gardnerella vaginalis* | *Streptococcus* spp | *Mobiluncus* spp | *Actinomyces* | *Fusobacterium* spp | *Candida* spp | *Candida albicans* | *Candida glabrata* | Cytomegalovirus | Herpes simple virus 1 |
| --- | --- | --- | --- | --- | --- | --- | --- | --- | --- | --- | --- | --- | --- | --- | --- | --- |
| *Ureaplasma parvum* |  | 3 | 1 | 0 | 0 | 0 | 11 | 2 | 0 | 1 | 0 | 11 | 2 | 0 | 1 | 0 |
| *Mycoplasma hominis* |  |  | 1 | 0 | 0 | 0 | 1 | 0 | 0 | 0 | 0 | 1 | 0 | 0 | 0 | 0 |
| *Ureaplasma urealyticum* |  |  |  | 0 | 0 | 0 | 1 | 0 | 0 | 0 | 0 | 0 | 0 | 0 | 1 | 0 |
| *Chlamydia trachomatis* |  |  |  |  | 0 | 0 | 0 | 0 | 0 | 0 | 0 | 0 | 0 | 0 | 1 | 0 |
| *Trichomonas vaginalis* |  |  |  |  |  | 0 | 1 | 0 | 0 | 0 | 0 | 0 | 0 | 0 | 0 | 0 |
| *Neisseria gonorrhoeae* |  |  |  |  |  |  | 0 | 0 | 0 | 0 | 0 | 0 | 0 | 0 | 0 | 0 |
| *Gardnerella vaginalis* |  |  |  |  |  |  |  | 3 | 3 | 0 | 2 | 7 | 0 | 0 | 1 | 0 |
| *Streptococcus* spp. |  |  |  |  |  |  |  |  | 0 | 0 | 0 | 3 | 0 | 0 | 0 | 0 |
| *Mobiluncus* spp. |  |  |  |  |  |  |  |  |  | 0 | 1 | 0 | 0 | 0 | 0 | 0 |
| *Actinomyces* |  |  |  |  |  |  |  |  |  |  | 0 | 2 | 0 | 1 | 1 | 0 |
| *Fusobacterium* spp. |  |  |  |  |  |  |  |  |  |  |  | 0 | 0 | 0 | 0 | 0 |
| *Candida* spp. |  |  |  |  |  |  |  |  |  |  |  |  | 0 | 1 | 1 | 0 |
| *Candida albicans* |  |  |  |  |  |  |  |  |  |  |  |  |  | 0 | 0 | 0 |
| *Candida glabrata* |  |  |  |  |  |  |  |  |  |  |  |  |  |  | 0 | 0 |
| Cytomegalovirus |  |  |  |  |  |  |  |  |  |  |  |  |  |  |  | 0 |
| Herpes simplex virus 1 |  |  |  |  |  |  |  |  |  |  |  |  |  |  |  |  |

*Table S5. Distribution of the different types of pathogens (bacterial. fungal. protozoan and viral) detected in endometrial samples and classified depending on the type of cycle (Natural LH or HRT) performed prior to the endometrial biopsy. The relative abundance was calculated concerning the total number of samples or relative to the total of samples within each group for the "Eubiosis" and "Dysbiosis" columns.*

|  | **Natural LH cycle** | | | | | | **HRT cycle** | | | | | |
| --- | --- | --- | --- | --- | --- | --- | --- | --- | --- | --- | --- | --- |
|  | **General** | | **Eubiosis** | | **Dysbiosis** | | **General** | | **Eubiosis** | | **Dysbiosis** | |
|  |  |  | **(% *Lactobacillus* ≥90%)** | | **(% *Lactobacillus* <90%)** | |  |  | **(% *Lactobacillus* ≥90%)** | | **(% *Lactobacillus* <90%)** | |
| **Type of pathogen** | **n** | **% vs sample total** | **n** | **% vs eu total** | **n** | **% vs dis total** | **n** | **% vs sample total** | **n** | **% vs eu total** | **n** | **% vs dis total** |
| ***Bacterial pathogens*** |  |  |  |  |  |  |  |  |  |  |  |  |
| *Ureaplasma parvum* | 50 | 13% | 13 | 19% | 37 | 11% | 94 | 8% | 18 | 10% | 76 | 8% |
| *Mycoplasma hominis* | 8 | 2% | 3 | 4% | 5 | 2% | 6 | 1% | 0 | 0% | 6 | 1% |
| *Ureaplasma urealyticum* | 3 | 1% | 2 | 3% | 1 | 0% | 13 | 1% | 0 | 0% | 13 | 1% |
| *Chlamydia trachomatis* | 2 | 1% | 0 | 0% | 2 | 1% | 2 | 0% | 1 | 1% | 1 | 0% |
| *Neisseria gonorrhoeae* | 0 | 0% | 0 | 0% | 0 | 0% | 1 | 0% | 0 | 0% | 1 | 0% |
| *Gardnerella vaginalis* | 38 | 10% | 2 | 3% | 36 | 11% | 118 | 10% | 5 | 3% | 113 | 12% |
| *Streptococcus* spp. | 5 | 1% | 0 | 0% | 5 | 2% | 26 | 2% | 1 | 1% | 25 | 3% |
| *Mobiluncus* spp. | 3 | 1% | 0 | 0% | 3 | 1% | 11 | 1% | 1 | 1% | 10 | 1% |
| *Actinomyces* | 7 | 2% | 1 | 1% | 6 | 2% | 18 | 2% | 2 | 1% | 16 | 2% |
| *Fusobacterium* | 1 | 0% | 0 | 0% | 1 | 0% | 2 | 0% | 0 | 0% | 2 | 0% |
| ***Fungal pathogens*** |  |  |  |  |  |  |  |  |  |  |  |  |
| *Candida* spp. | 37 | 9% | 7 | 10% | 30 | 9% | 89 | 8% | 5 | 3% | 84 | 9% |
| *Candida albicans* | 3 | 0% | 0 | 0% | 3 | 1% | 12 | 0% | 5 | 3% | 7 | 1% |
| *Candida glabrata* | 2 | 0% | 0 | 0% | 2 | 1% | 0 | 0% | 0 | 0% | 0 | 0% |
| ***Viral pathogens*** |  |  |  |  |  |  |  |  |  |  |  |  |
| Cytomegalovirus | 5 | 1% | 2 | 3% | 3 | 1% | 17 | 1% | 3 | 2% | 14 | 1% |
| Herpes simplex virus 1 | 0 | 0% | 0 | 0% | 0 | 0% | 1 | 0% | 0 | 0% | 1 | 0% |
| ***Protozoo*** |  |  |  |  |  |  |  |  |  |  |  |  |
| *Trichomonas vaginalis* | 1 | 0% | 0 | 0% | 1 | 0% | 1 | 0% | 0 | 0% | 1 | 0% |

*Figure S1. Flow chart of patients who had more than one biopsy performed.*

*Figure S2. Number of samples with increasing Lactobacillus percentage in the 1^st^ and the 2^nd^ biopsy of the same patients. The 2^nd^ biopsy was performed after treatment with antibiotics and/or probiotics.*

Sample number

| Lactobacillus % | 1st biopsy | 2nd biopsy |
| --- | --- | --- |
| 0-10 | 33 | 20 |
| 10-20 | 8 | 9 |
| 20-30 | 3 | 5 |
| 30-40 | 2 | 6 |
| 40-50 | 5 | 5 |
| 50-60 | 3 | 4 |
| 60-70 | 1 | 2 |
| 70-80 | 7 | 4 |
| 80-90 | 3 | 6 |
| 90-100 | 12 | 16 |

*Table S6. Description of the pathogens detected in patients with multiple biopsies and a pathogenic result in the first biopsy. The antimicrobial treatment that was used in each case is detailed. as well as the type of profile (pathogenic/non pathogenic).*

|  | **1st biopsy** | | | **2nd biopsy** | | | **3rd biopsy (if aplicable)** | | |
| --- | --- | --- | --- | --- | --- | --- | --- | --- | --- |
| **Patient** | **Classification (Pathogenic/ non pathogenic)** | **Detected pathogenic species** | **Antimicrobial treatment** | **Classification (Pathogenic/ non pathogenic)** | **Detected pathogenic species** | **Antimicrobial treatment** | **Classification (Pathogenic/non pathogenic)** | **Detected pathogenic species** | **Antimicrobial treatment** |
| *1* | Pathogenic | *Mycoplasma hominis* | NC | Pathogenic | *Candida* spp. | NC | NA |  |  |
| *2* | Pathogenic | *Mobiluncus* | Amoxicillin/clavulanic acid | Non pathogenic | ND |  | NA |  |  |
| *3* | Pathogenic | *Actinomyces* | Doxycycline | Pathogenic | *Ureaplasma parvum* | Levofloxacin | Non pathogenic |  |  |
| *4* | Pathogenic | *Ureaplasma parvum* | Azithromycin | Non pathogenic | ND |  | NA |  |  |
| *5* | Pathogenic | *Mobiluncus* | Dequalinium chloride | Non pathogenic | ND |  | NA |  |  |
| *6* | Pathogenic | *Actinomyces* | Doxycycline | Non pathogenic | ND |  | NA |  |  |
| *7* | Pathogenic | *Ureaplasma parvum. Mycoplasma hominis* | Doxycycline, dequalinium chloride | Non pathogenic | ND |  | NA |  |  |
| *8* | Pathogenic | *Atopobium. Corynebacterium* | Dequalinium chloride, ciprofloxacin | Non pathogenic | ND |  | NA |  |  |
| *9* | Pathogenic | *Ureaplasma parvum* | Azithromycin | Pathogenic | *Ureaplasma parvum* | Doxycycline | Non pathogenic |  |  |
| *10* | Pathogenic | *Actinomyces* | Doxycycline | Non pathogenic | ND |  | NA |  |  |
| *11* | Pathogenic | *Mobiluncus. Ureaplasma urealyticum. Ureaplasma parvum* | NC | Non pathogenic | ND |  | NA |  |  |
| *12* | Pathogenic | Cytomegalovirus | Serological monitoring (no treatment) | Non pathogenic | ND |  | NA |  |  |
| *13* | Pathogenic | *Actinomyces* | Doxycycline | Non pathogenic | ND |  | NA |  |  |
| *14* | Pathogenic | *Actinomyces* | Doxycycline | Non pathogenic | ND |  | NA |  |  |
| *15* | Pathogenic | *Ureaplasma parvum. Gardnerella vaginalis* | Doxycycline (partner untreated) | Pathogenic | *Ureaplasma parvum* | Doxycycline (partner treated) | Pathogenic | Cytomegalovirus | Untreated |
| *16* | Pathogenic | *Mobiluncus* | Amoxicillin/clavulanic acid | Non pathogenic | ND |  | NA |  |  |
| *17* | Pathogenic | *Ureaplasma parvum* | Doxycycline | Non pathogenic | ND |  | NA |  |  |
| *18* | Pathogenic | *Gardnerella vaginalis* | Dequalinium chloride | Non pathogenic | ND |  | NA |  |  |
| *19* | Pathogenic | *Ureaplasma parvum* | Doxycycline | Non pathogenic | ND |  | NA |  |  |
| *20* | Pathogenic | *Ureaplasma parvum* | Doxycycline | Non pathogenic | ND |  | NA |  |  |
| *21* | Pathogenic | *Ureaplasma parvum* | Doxycycline | Non pathogenic | ND |  | NA |  |  |
| *22* | Pathogenic | *Ureaplasma parvum. Atopobium* | Doxycycline, dequalinium chloride | Non pathogenic | ND |  | NA |  |  |
| *23* | Pathogenic | *Chlamydia trachomatis*. Cytomegalovirus | Doxycycline, Valganciclovir | Non pathogenic | ND |  | NA |  |  |
| *24* | Pathogenic | *Actinomyces* | Doxycycline | Non pathogenic | ND |  | NA |  |  |
| *25* | Pathogenic | *Ureaplasma parvum* | Azithromycin | Non pathogenic | ND |  | NA |  |  |
| *26* | Pathogenic | *Gardnerella vaginalis* | Dequalinium chloride | Non pathogenic | ND |  | NA |  |  |
| *27* | Pathogenic | *Atopobium. Enterococcus. Enterobacteriaceae* | Dequalinium chloride | Non pathogenic | ND |  | NA |  |  |
| *28* | Pathogenic | *Ureaplasma parvum* | Doxycycline | Non pathogenic | ND |  | NA |  |  |
| *29* | Pathogenic | *Ureaplasma parvum* | Doxycycline | Non pathogenic | ND |  | NA |  |  |
| *30* | Pathogenic | *Ureaplasma parvum* | Doxycycline | Pathogenic | *Gardnerella vaginalis* | Dequalinium chloride | NA |  |  |
| *31* | Pathogenic | *Ureaplasma parvum* | Doxycycline | Non pathogenic | ND |  | NA |  |  |
| *32* | Pathogenic | *Ureaplasma parvum* | Doxycycline | Non pathogenic | ND |  | NA |  |  |
| *33* | Pathogenic | *Ureaplasma parvum. Atopobium* | Doxycycline, dequalinium chloride | Pathogenic | *Streptococcus* spp. | Ampicillin | NA |  |  |
| *34* | Pathogenic | *Ureaplasma parvum* | Doxycycline | Non pathogenic | ND |  | NA |  |  |
| *35* | Pathogenic | *Ureaplasma parvum* | Doxycycline, dequalinium chloride | Pathogenic | *Ureaplasma parvum* | NC | NA |  |  |
| *36* | Pathogenic | *Ureaplasma urealyticum* | NC | Pathogenic | *Ureaplasma urealyticum* | NC | NA |  |  |
| *37* | Pathogenic | *Gardnerella vaginalis* | Dequalinium chloride | Non pathogenic | ND |  | NA |  |  |
| *38* | Pathogenic | *Atopobium. Prevotella. Actinomyces* | NC | Pathogenic | *Gardnerella vaginalis. Streptococcus* spp. | NC | NA |  |  |
| *39* | Pathogenic | *Atopobium. Ureaplasma* | Doxycycline | Non pathogenic | ND |  | NA |  |  |
| *40* | Pathogenic | *Ureaplasma parvum* | Doxycycline | Non pathogenic | ND |  | NA |  |  |
| *41* | Pathogenic | *Actinomyces* | Doxycycline | Non pathogenic | ND |  | NA |  |  |
| *42* | Pathogenic | *Corynebacterium. Atopobium* | Dequalinium chloride | Pathogenic | *Streptococcus* spp. | Ampicillin | NA |  |  |
| *43* | Pathogenic | *Ureaplasma parvum. Gardnerella vaginalis* | Levofloxacin | Non pathogenic | ND |  | NA |  |  |
| *44* | Pathogenic | *Ureaplasma parvum* | Doxycycline | Non pathogenic | ND |  | NA |  |  |
| *45* | Pathogenic | *Mobiluncus* | Amoxicillin/clavulanic acid | Non pathogenic | ND |  | NA |  |  |
| *46* | Pathogenic | *Ureaplasma parvum* | Doxycycline | Non pathogenic | ND |  | NA |  |  |
| *47* | Pathogenic | *Gardnerella vaginalis* | Dequalinium chloride | Non pathogenic | ND |  | NA |  |  |
| *48* | Pathogenic | *Ureaplasma parvum* | Doxycycline | Non pathogenic | ND |  | NA |  |  |
| *49* | Pathogenic | *Gardnerella vaginalis* | Dequalinium chloride | Non pathogenic | ND |  | NA |  |  |
| *50* | Pathogenic | *Atopobium vaginae. Gardnerella vaginalis. Ureaplasma parvum* | Azithromycin | Pathogenic | *Ureaplasma parvum* | Doxycycline | NA |  |  |
| *51* | Pathogenic | *Ureaplasma parvum. Gardnerella vaginalis* | Doxycycline, dequalinium chloride | Non pathogenic | ND |  | NA |  |  |
| *52* | Pathogenic | *Atopobium vaginae. Gardnerella vaginalis* | Dequalinium chloride | Non pathogenic | ND |  | NA |  |  |
| *53* | Pathogenic | *Ureaplasma parvum* | Levofloxacin | Non pathogenic | ND |  | NA |  |  |
| *54* | Pathogenic | *Gardnerella vaginalis* | Dequalinium chloride, clindamycin | Non pathogenic | ND |  | NA |  |  |
| *55* | Pathogenic | *Corynebacterium. Staphylococcus* | Ciprofloxacin | Non pathogenic | ND |  | NA |  |  |
| *56* | Pathogenic | *Corynebacterium* | Ciprofloxacin | Non pathogenic | ND |  | NA |  |  |
| *57* | Pathogenic | *Gardnerella vaginalis* | Metronidazole, dequalinium chloride | Pathogenic | *Mobiluncus* spp. | Amoxicillin | Non pathogenic |  |  |
| *58* | Pathogenic | *Mycoplasma hominis. Ureaplasma parvum* | Doxycycline | Non pathogenic | ND |  | NA |  |  |
| *59* | Pathogenic | *Ureaplasma parvum* | Azithromycin | Pathogenic | *Ureaplasma parvum* | Doxycycline | Pathogenic | Actinomyces | Doxycycline |
| *60* | Pathogenic | *Atopobium vaginae. Streptococcus* spp. | Only probiotic treatment | Non pathogenic | ND |  | NA |  |  |
| *61* | Pathogenic | *Actinomyces* | Doxycycline | Non pathogenic | ND |  | NA |  |  |
| *62* | Pathogenic | *Gardnerella vaginalis* | Dequalinium chloride | Pathogenic | *Gardnerella vaginalis* | Dequalinium chloride | NA |  |  |
| *63* | Pathogenic | *Mobiluncus. Gardnerella vaginalis* | Amoxicillin/clavulanic acid, dequalinium chloride | Non pathogenic | ND |  | NA |  |  |
| *64* | Pathogenic | *Streptococcus* spp. | NC | Non pathogenic | ND |  | NA |  |  |
| *65* | Pathogenic | *Gardnerella vaginalis* | Dequalinium chloride | Non pathogenic | ND |  | NA |  |  |
| *66* | Pathogenic | *Atopobium vaginae. Ureaplasma parvum* | Azithromycin, dequalinium chloride | Pathogenic | *Ureaplasma parvum* | Doxycycline | NA |  |  |
| *67* | Pathogenic | *Gardnerella vaginalis. Trichomonas vaginalis* | NC | Pathogenic | *Ureaplasma parvum* | NC | Non pathogenic |  |  |
| *68* | Pathogenic | Cytomegalovirus | NC | Non pathogenic | ND |  | NA |  |  |
| *69* | Pathogenic | Cytomegalovirus | Valganciclovir | Non pathogenic | ND |  | NA |  |  |
| *70* | Pathogenic | *Actinomyces*. Cytomegalovirus | Doxycycline, Valganciclovir | Pathogenic | *Gardnerella vaginalis* | Dequalinium chloride | NA |  |  |
| *71* | Pathogenic | *Actinomyces. Candida glabrata* | NC | Non pathogenic | ND | NC | NA |  |  |

*NC: not confirmed; ND: not detected; NA: not applicable

*Table S7. Distribution of the different profiles regarding Lactobacillus dominance (eubiosis/dysbiosis) and presence of pathogens in patients with different clinical indications.*

|  | **All patients** | | **Implantation failure (IF)** | | **Recurrent implantation failure (RIF)** | | **Miscarriage** | | **Other indications** | |  |
| --- | --- | --- | --- | --- | --- | --- | --- | --- | --- | --- | --- |
|  | **n** | **Percentage** | **n** | **Percentage** | **n** | **Percentage** | **n** | **Percentage** | **n** | **Percentage** | |
| TOTAL | 1619 | 100.00% | 853 | 100.00% | 289 | 100.00% | 201 | 100.00% | 276 | 100.00% | |
| EUBIOSIS | 271 | 16.74% | 140 | 16.41% | 46 | 15.92% | 38 | 18.91% | 47 | 17.03% | |
| *Non-pathogenic eubiosis* | 202 | 12.48% | 102 | 11.96% | 36 | 12.46% | 28 | 13.93% | 36 | 13.04% | |
| *Pathogenic eubiosis* | 69 | 4.26% | 38 | 4.45% | 10 | 3.46% | 10 | 4.98% | 11 | 3.99% | |
| DYSBIOSIS | 1348 | 83.26% | 713 | 83.59% | 243 | 84.08% | 163 | 81.09% | 229 | 82.97% | |
| *Non-pathogenic dysbiosis* | 880 | 54.35% | 464 | 54.40% | 158 | 54.67% | 102 | 50.75% | 156 | 56.52% | |
| *Pathogenic dysbiosis* | 468 | 28.91% | 249 | 29.19% | 85 | 29.41% | 61 | 30.35% | 73 | 26.45% | |
